# Use of Mining Waste to Produce Ultra-High-Performance Fibre-Reinforced Concrete

**DOI:** 10.3390/ma13112457

**Published:** 2020-05-28

**Authors:** Jesús Suárez González, Iñigo Lopez Boadella, Fernando López Gayarre, Carlos López-Colina Pérez, Miguel Serrano López, Flavio Stochino

**Affiliations:** 1 Poliytechnic School of Engineering, Campus de Viesques, University of Oviedo, 33203 Gijón, Spain; inigo2208@hotmail.com (I.L.B.); gayarre@uniovi.es (F.L.G.); lopezpcarlos@uniovi.es (C.L.-C.P.); serrano@uniovi.es (M.S.L.); 2 Department of Civil, Environmental and Architectural Engineering, University of Cagliari, 09100 Cagliari, Italy; fstochino@unica.it

**Keywords:** mining waste, ultra-high performance fibres reinforced concrete, compressive strength, flexural strength

## Abstract

This research work analyses the influence of the use of by-products from a fluorite mine to replace the fine fraction of natural aggregates, on the properties of Ultra-High-Performance Fibre-Reinforced Concrete (UHPFRC). Replacing natural aggregates for different kinds of wastes is becoming common in concrete manufacturing and there are a number of studies into the use of waste from the construction sector in UHPFRC. However, there is very little work concerning the use of waste from the mining industry. Furthermore, most of the existing studies focus on granite wastes. So, using mining sand waste is an innovative alternative to replace natural aggregates in the manufacture of UHPFRC. The substitutions in this study are of 50%, 70% and 100% by volume of 0–0.5 mm natural silica sand. The results obtained show that the variations in the properties of consistency, compressive strength, modulus of elasticity and tensile strength, among others, are acceptable for substitutions of up to 70%. Therefore, fluorite mining sand waste is proved to be a viable alternative in the manufacturing of UHPFRC.

## 1. Introduction

In 1973, the overexploitation of natural resources and environmental degradation due to the waste generated in industry, mining, etc. motivated the European Union to launch its Environment Action Program. This program is currently in its seventh edition. The latest resolutions, passed in 2013, prioritize increasingly efficient use of resources to make the European Union an intelligent, sustainable and inclusive economy. Construction is one of the most affected sectors, so the use of waste as a resource is currently a priority in the research lines of many institutions in this sector, particularly in relation to the manufacturing of concrete.

Ultra High Performance Concrete (UHPC) emerged in the early 1990s, when P. Richard [1] developed a new type of concrete that offered a compressive strength of over 200 MPa, a bending strength above 40 MPa, and some ductility. In 2002 the first recommendations for the structural use of these concretes ware published in France [2]. In 2005, noteworthy research work was carried out in Germany, building the groundwork for the basic knowledge needed to develop a reliable, economically feasible UHPC [3]. UHPC has been developed further in recent decades and is valued for its high mechanical strength and very low porosity.

The incorporation of short steel fibres in these concretes improves the performance of the structures against flexotraction, controls and reduces cracking. These concretes are called Ultra-High-Performance Fibre-Reinforced Concrete (UHPFRC). Abbas et al. [4] reported that the incorporation of short steel fibres, increases the load against the first crack and the maximum load, as well as making it possible to double or triple the tensile strength of UHPC. Although the Association Française de Génie Civil considers UHPC as that having a compressive strength higher than 150 MPa [2], it is very common to classify concrete with a compressive strength of over 100MPa as UHPC. In this regard, F. Canovas qualifies concrete as having very-high strength when it has a compressive strength higher than 90 MPa and as having ultra-high strength when its compressive strength is higher than 125 MPa at 28 days [5].

Ecologically, however, UHPC has problems in terms of sustainability, since it uses very fine fractions of natural aggregates, especially silica sands, which require significant energy for grinding. Therefore, there is a need to find and supply alternative materials to avoid or reduce their use. The use of recycled materials or construction by-products as a total or partial substitution of natural aggregates in the search for a more sustainable UHPC is currently being researched. Ambily el al. [6] study the use of copper slags as an alternative to the finest aggregate in UHPC. With 100% substitution of the fine aggregate, variations in flexural strength are negligible, and despite the slight reported loss of compressive strength, the final values are above 150 MPa. Zhu et al. [7] and Zhao et al. [8] use iron tailings as an alternative to natural aggregate. They observed that up to 40% substitution, the mechanical properties of the UHPC were coMParable to those of the control concrete. In addition, Zhao et al. [8] showed that if the maximum size of the iron tailings was under 1.20 mm, a substitution of 60% is viable.

Glass waste is another type of material that can be used to substitute aggregates. Yang et al. [9] and Soliman and Tagnit-Hamou [10] have shown that using recycled glass to replace the natural sand gives promising mechanical properties in UHPC. For a ratio of 50% substitution, the slump and the compressive strength are coMParable to the control UHPC mix [10]. Following this same line of research, Zegardlo et al. [11] have shown that ceramic waste is another alternative to natural aggregate in the manufacture of UHPC. The results show that the compressive strength improves by 24% and the tensile strength rises to 34%.

The abovementioned studies focus on the use of waste from the construction sector. However, there are only a few studies that focus on incorporating waste from the extractive industry in the manufacture of concrete, specifically HPC and UHPC. The studies in which granite cutting waste is used as a substitution for fine aggregate in UHPC is relevant in this area. Sarbjeet et al. [12] showed that a substitution of 40% of the aggregate had a positive iMPact on the flexural strength while for a ratio of 70% the results were coMParable to those of the control concrete. López Boadella et al. [13] observed an increase in flexural strength and tensile strength by substituting 35% of micronized quartz with fine granite cutting waste. Similar results were provided by Kala [14] who found that the incorporation of granite fines as a partial replacement for sand has a positive effect on the properties of HPC in relation to compressive strength, flexural strength and tensile strength.

Mining, which is one of the greatest waste-generating activities [15,16] is the focus of this research. This a study of the feasibility of the utilization of by-products from a fluorite (CaF_2_) mine as a replacement for natural aggregates in the manufacture of UHPFRC. This silica sand comes from the mine’s washing installations. Once treated and washed, it could be used in the manufacture of UHPC. However, it cannot be used in conventional concrete due to the high percentage of fines. To the best of the authors’ knowledge, there are no previous studies on the use of this type residue. The studies than come close are those already mentioned, which refer to granite fines. So, the main objective of this research is the innovative use of this by-product as a substitute for natural aggregates in the manufacture of UHPFRC.

## 2. Experimental Study

### 2.1. Description of the Materials Used

**Figure 1:**
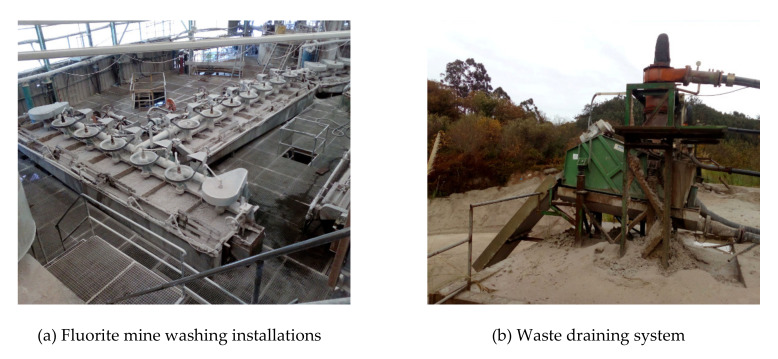
Obtaining residual mine sand.

Type I 42.5 R/SR Ordinary Portland Cement (OPC, Lafarge-Holcim, Madrid, Spain) was used. As additions for the mix, two materials were used: densified silica fume (Elkem Microsilica® 940, Elkem, Barcelona, Spain) with a 0.15 µm average particle size and micronized quartz with a maximum particle size of 40 µm. The steel fibres (Arcelor Mittal, Asturias, Spain) used in the research had a diameter of 0.2 mm and a length of 13 mm. As natural aggregates, two types of siliceous sands (Sílices La Cuesta, Asturias, Spain) with granulometric fractions 0–0.5 mm and 0.5–1.6 mm were used. In order to achieve optimum workability, a polyxycarboxylate superplasticizer was used. (Sika ViscoCrete-225 Powder, Sika, Madrid, Spain). Finally, as a substitute for the finest fraction of the sand, waste mining sand (WMS) (Minersa, Ribadesella, Spain), which is generated in the process of obtaining fluoride or fluorite spar in fluorite mines, was used (Figure 1a,b).

Table 1 shows a summary of the main properties of all the materials used. The greatest amount of fine particles present in the WMS is in the sand equivalent test.

The chemical composition of the WMS is determined by X-ray fluorescence. The results obtained are shown in Table 2, where L.O.I stands for Loss on Ignition. The second most significant component is CaO, at 10%. The percentages of Al_2_O_3_ and Fe_2_O_3_ are approximately 1.5%, higher than those of the 0–0.5 mm silica sand.

**Figure 2:**
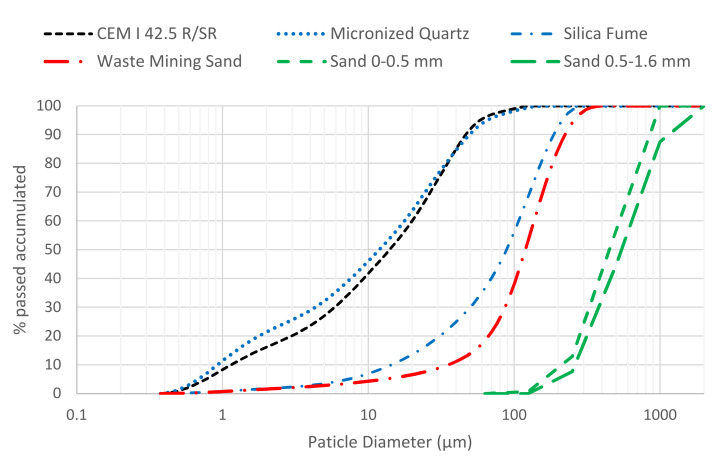
Grading curves of the materials.

Figure 2 shows the particles size distribution of the materials used. A difference between the granulometry of the 0–0.3 mm WMS, and the fraction of 0–0.5 mm silica sand which it substitutes, can be seen. The granulometric distribution of silica fume is a consequence of its densification. Its maximum particle size is 0.15 μm.

### 2.2. Mix Design

To develop this research, the starting point was a self-coMPacting reference concrete with a compressive strength of more than 117 MPa. From this control mix, the experimental program was developed with replacements of 50%, 70% and 100% of 0–0.5 mm of silica sand for the same volume of WMS. Substitutions below 50% were not done as the results obtained for these percentages were satisfactory.

Table 3 shows the proportions of each of the UHPFRC mixes manufactured.

**Figure 3:**
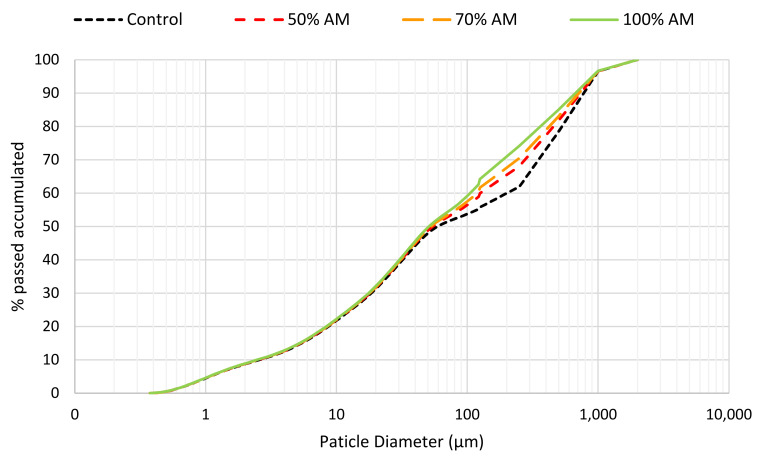
Grading curves of UHPFRC.

Figure 3 shows the grading curves of the total aggregate of the different UHPFRC mixes. As the percentage of substitution of the finest fraction of the sand for WMS increases, the percentage of particles with a size between 50 µm and 300 µm increases. This increase generates grading curves with a more continuous progression, given that the depression that occurs in the range between 50 µm and 1 mm for the control concrete is corrected. Because of this, the curves resulting from the use of mine sand are more similar to the ideal packing curve proposed by authors such as Andreasen or Funk and Dinger [17].

### 2.3. Development of the Experimental Program

The mix procedure is as follows: first the two parts of silica sand and/or the WMS are introduced in the mixer, followed by the micronized quartz, the silica fume and finally the cement. They are mixed for 30 s and then the whole amount of water is poured in. Mixing continues for 2 min more, after which the superplasticizer additive and the steel fibres are added. The whole mixing process ends after 25 min.

Nine specimens were manufactured for each mix, according to the specifications of standard UNE-EN 12390-1 [18]: three Ø15 × 30 cm cylindrical specimens, three 10 × 10 × 10 cm cubic specimens and three 10 × 10 × 40 cm prismatic specimens. The specimens were cured in a humid chamber at a temperature of 20 ± 2 °C and a relative humidity of 95% for 28 days following the specifications of the UNE-EN 12390-2 standard [19].

The following properties were measured: the consistency of fresh UHPFRC according to the NF P 18-470 standard [20], the density of hardened UHPFRC following the UNE-EN 12390-7 standard [21], the modulus of elasticity (UNE- EN 12390-13) [22], compressive strength (UNE-EN 12390-3) [23], flexural strength (NF P 18-470) [20] and tensile strength (NF P standard 18-470) [20].

Table 4 shows a summary of the results obtained in this study. All values, except slump, correspond to the average value of the three results obtained in each of the tests performed.

## 3. Analysis of Results

### 3.1. Consistency of Fresh UHPFRC

**Figure 4:**
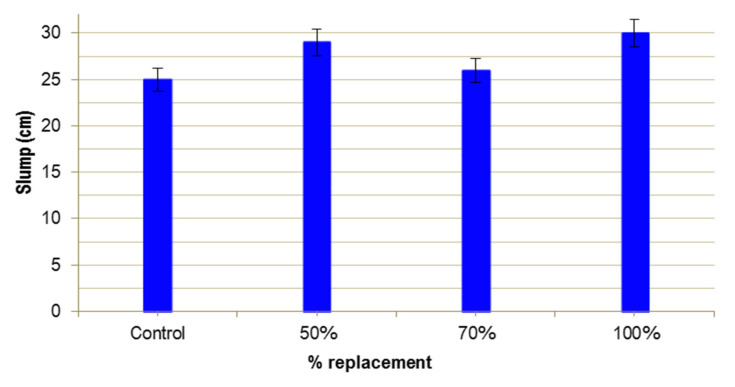
Slump of UHPFRC.

Figure 4 shows the results obtained. In the three mixes there is an increase in the consistency of fresh UHPFRC, of between 4% and 25%. This improvement may be related with the variation in the granulometry of WMS (0–0.3 mm), in relation to the fraction of substituted silica sand (0–0.5 mm). It may also be related to the variation experienced by the grading curve of the mixtures in the range 0.1–0.5 mm when introducing the mine sand, since a more continuous distribution of the particle size is observed. This will improve packaging and reduce water requirements [24].

Differences between these results and those obtained by other authors can be explained by the characteristics of the type of waste used. Soliman and Tagnit-Hamou [10] observed an improvement in slump flow by incorporating glass powder, with slightly larger average particle diameter, as partial or total substitution of quartz sand. Similar results were obtained by Pyo et al. [25] when substituting the finest silica sand with waste generated in a tungsten mine. For substitutions of 50% and 100% there is an improvement in the flow of fresh concrete greater than 30%. However, Kou [26] and Zhao et al. [8] observe a decrease in flowability in their tests when using other types of waste. Kou [26] shows a loss in the flowability of UHPFRC when discarded fly ash is incorporated as a substitution for silica sand. Kou attributes this loss of flowability to a greater amount of fines present in fly ash. Zhao et al. [8] observed that substituting 100% of the natural aggregate with iron ore tailings produced a significant decrease in the flowability of the UHPC due to the more angular and irregular shape of the tailings.

### 3.2. Density of the Hardened UHPFRC

**Figure 5:**
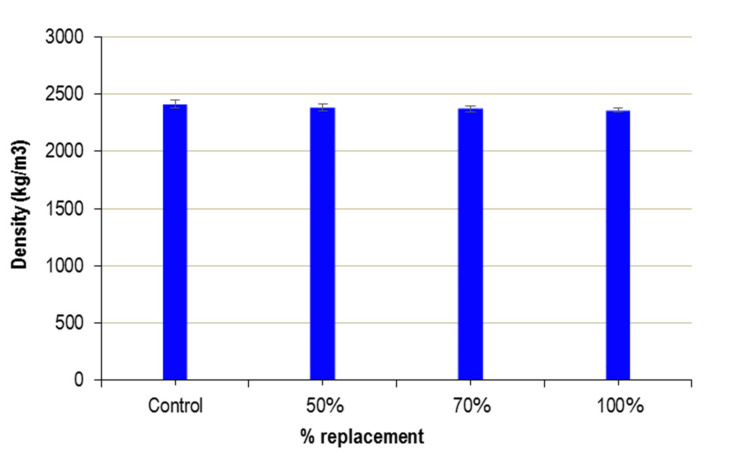
Density of UHPFRC.

Figure 5 shows the values obtained for the different substitution percentages. As the percentage of WMS increases, there is a minimal density reduction, around 2% for a 100% substitution. These variations in density are so slight as to be within the range of variability of the results obtained, as shown in the error bars in Figure 5. Therefore, the use of WMS has a negligible influence on the density of hardened UHPFRC.

### 3.3. Compressive Strength

**Figure 6:**
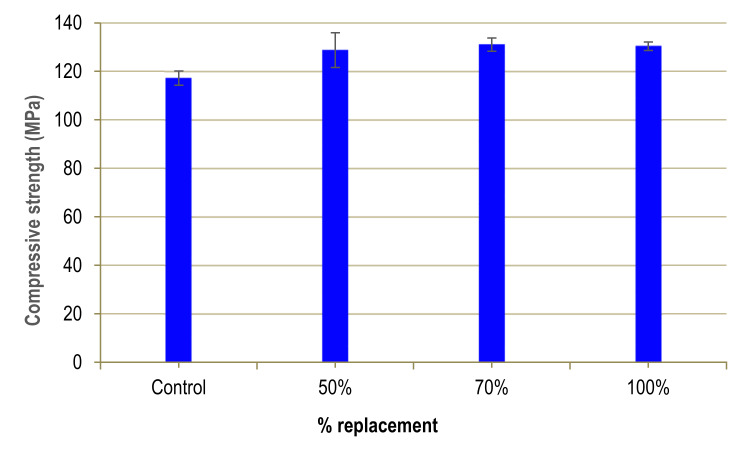
Compressive strength of UHPFRC.

There is an increase in strength for all degrees of substitution, as shown in Figure 6. This may be due to the difference between the maximum aggregate size of silica sand (0.5 mm) and that of sand from the fluorite mine (0.3 mm) since, especially in cement-rich concrete. The smaller the maximum particle size, the greater the compressive strength of the concrete [27,28,29].

These increases in the compressive strength of concrete are in line with the results obtained by Zegardlo, [11] who obtained an increase in strength of 24.7% when using ceramic waste as an alternative to natural aggregate. He attributes this improvement to the formation of mechanical hooks in the interfacial transition zone (ITZ), which generate better adhesion with the cement paste and greater strength. Similar results were obtained by Zhu [7] who observed an increase in compressive strength by incorporating 0–1.20 mm iron ore tailings to substitute 0–4.75 mm siliceous sands, independently of the percentage of substitution. However, other authors obtain less favourable results, depending on the waste used: Soliman and Tagnit-Hamou report a loss of compressive strength of 15% for a 100% substitution in their study using glass waste as an alternative to quartz sand [10].

## 4. Elasticity Modulus

**Figure 7:**
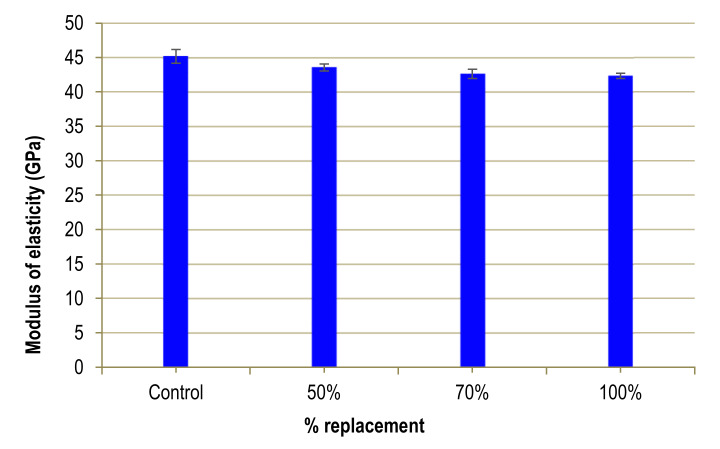
Modulus of Elasticity of UHPFRC.

Figure 7 displays the effect of WMS on the elasticity modulus of the UHPFRC. A slight and progressive decrease in the modulus of elasticity values can be seen as the substitution percentage increases. However, this difference is small, not exceeding 6%.

This low influence on the modulus of elasticity is also reflected in the results obtained by Alsalman [30], when substituting sand with fly ash. According to the author, this may be due to an excess of fly ash, which makes it impossible for 100% of these particles to be hydrated. This counteracts the positive effect of hydrated ash.

Gonzalez-Corominas and Etxeberria [31] also observed a decrease in the modulus of elasticity (around 5%), although their study refers to low substitution percentages (less than 30%) and uses ceramic waste to manufacture high performance concrete (HPC). They relate this reduction to the lower density of the concrete derived from using ceramic waste.

### 4.1. Flexural Strength

**Figure 8:**
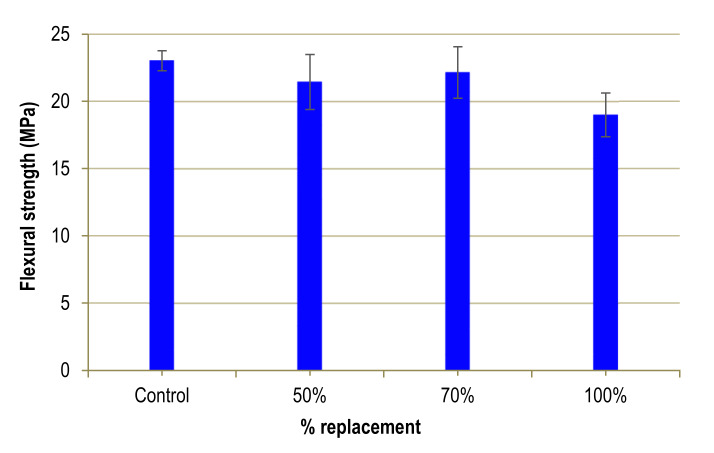
Flexural strength of UHPFRC.

The results regarding flexural strength, shown in Figure 8**,** are stable up to 70% substitution, when they rise slightly. In general, the trend is declining and drops to 17% for a substitution of 100%. For lower percentages the variation is smaller, less than 7%. The variability of the results may be due to the randomness of the distribution of steel fibres.

The explanation may be in the inferior quality of mine sand, since it has 10% CaO, as opposed to the natural sand it substitutes, which is almost 100% SiO_2_. This could counteract the advantageous results produced by the improvement in the grading curve for high percentage substitutions, because of the lower mechanical resistance of the mine sand. In general, the result is similar to that obtained by Zhao et al. [8] and Zhu et al. [7] who use iron ore tailings as an alternative to natural aggregate. The concrete loses 18% of its flexural strength when 100% of the natural aggregate is substituted, while for the rest of the percentages the variations are very small. These results are also similar to those obtained by Taha [32], who uses glass waste as an alternative to natural aggregate in UHPC without fibres, although the results of flexural strength are not coMParable due to the lack of fibres. However, the results obtained by this author show a decrease in flexural strength of 21%, when 100% of the fine aggregate is substituted. Taha considers that this loss of strength may be a consequence of small cracks present in the particles of the glass waste, or the presence of organic materials, which can degrade over time, creating hollows in the microstructure of the concrete.

### 4.2. Tensile Strength

**Figure 9:**
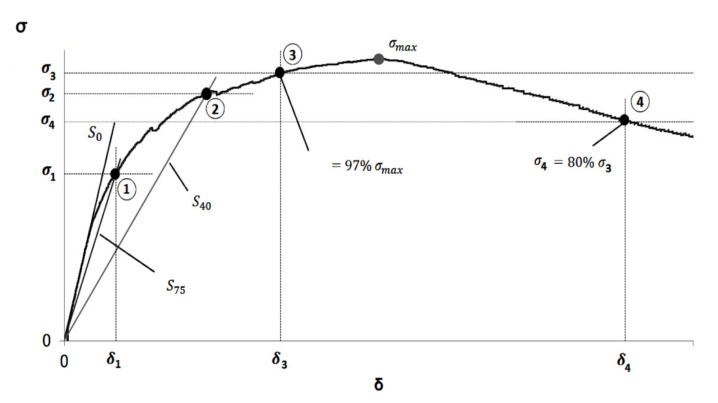
Stress–strain curves and key points.

In order to determinate the tensile strength of UHPFRC the stress-strain curves from the flexural tests have been taken as the starting point as seen in Figure 9.

From the curve obtained, the key points [33] necessary to determine the tensile behaviour were determined, using the following points:

Point 1 is the intersection of the curve test with a line of a slope equal to 0.75 of the slope of the elastic zone of the curve.

Point 2 is the intersection of the curve test with a line of a slope equal to 0.40 of the slope of the elastic zone of the curve.

Point 3 is the point of the ascending zone of the curve, with 97% of the highest stress.

Based on these points, the tensile strength of the UHPFRC can be obtained by the following equations [33]:

(1) E=2.40 h m

(2) $$f_{t}=\frac{\sigma_{75}}{1,63}{\left(\frac{\sigma_{75}}{\sigma_{40}}\right)}^{0.19}$$

(3) $$\varepsilon_{t,u}=\frac{f_{t}}{E}\left(7.65\;\frac{\delta_{loc}}{\delta_{75}}-10.53\right)$$

(4) $$\varepsilon_{t,\;el}=f_{t}/E$$

(5) $$\alpha =\varepsilon_{t,u}/\varepsilon_{t,el}$$

(6) $$f_{t,u}=\alpha^{-0.18}\left(2.46\;\frac{\sigma_{loc}}{\sigma_{75}}-1.76\right)f_{t}$$

Where the pair of values (*σ_75_*, *δ_75_*), (*σ_40_*, *δ_40_*), and (*σ_loc_*, *δ_loc_*) correspond respectively with points 1, 2 and 3 in the curve of Figure 9. Equation (1) represents the value of elasticity modulus (*E*) in function of the slope (*m*) of the elastic region of the stress-strain curve and the thickness (*h*) of the specimen in mm. Equation (2) refers to the crack resistance (*f_t_*) of the matrix reinforced with fibres, Equation (3) calculates the peak deformation (*ε_t,u_*), Equations (4) and (5) refers to the standardised parameters *ε_t,el_* and α, and Equation (6) represents the value of the ultimate tensile strength (*f_t,u_*) in function of the parameters previously defined.

**Figure 10:**
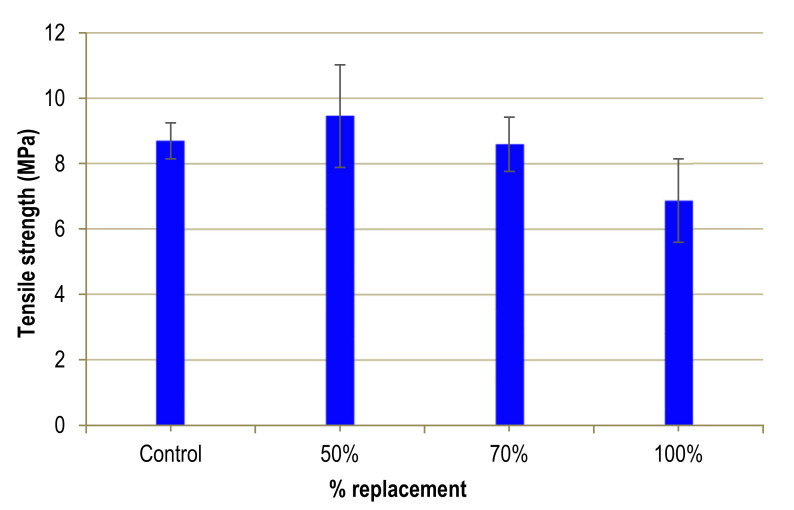
Tensile strength of UHPFRC.

The resulting tensile strengths can be seen in Table 4 and Figure 10. An initial increase in tensile strength values is followed by a decrease for high substitution percentages. For a 50% substitution, an increase in resistance of 9.5 MPa is achieved, which represents an increase of 9%, whereas for a 100% substitution, there is a loss of 20%. For a substitution of 70%, there is hardly any variation with respect to the control UHPFRC. As the error bars show, a high variability of the experimental results obtained for each substitution percentage is clear. This variation may be due to the random distribution of the fibres in the UHPFRC.

The tensile strength results are similar to those of flexural strength, with an improvement for low substitution percentages followed by a drop that reaches 20% for a 100% substitution. The possible explanation, as in flexural strength, is the inferior quality of the mine sand used. The result obtained is similar to that of Taha [32] when analysing the influence of substituting glass waste for sand in the UHPC without fibres, although pointing out that the absence of fibres is an important factor. This author observes very little variation for 50% substitution, while for 100%, he reports a loss of tensile strength of 5%. As mentioned in the previous section, Taha considers that this loss may be due to small cracks present in the particles of the glass waste.

## 5. Conclusions

In this research, two main findings have been identified, which could explain the results obtained in the laboratory tests. The first is that the grading curve of the mixtures shows a more continuous distribution of the particle size, which improves packaging and reduce water requirements. The second is that the quality of mining sand is slightly inferior given that it contains 10% CaO as opposed to the natural sand it substitutes, which is almost 100% SiO_2_. The overall consequence is that the improvement in packaging leads to an improvement in mechanical properties up approximately 70% of replacement, at which point the lower quality of waste mining sand counteracts the advantages of the better packaging.

In view of the results obtained in this work, it can be established that waste sand from fluorite mines (WMS) is a viable alternative as a partial substitution of silica sand in the manufacturing of Ultra High Performance Fibre Reinforced Concrete (UHPFRC). The most relevant conclusions are the following:

The incorporation of WMS improves the consistency of fresh UHPFRC due, probably, to its smaller particle size generating a lubrication effect of the mixture.

The density of UHPFRC with WMS is very similar to the density of the control concrete. The variations are very small, less than 2.5%, for any percentage of substitution.

The improved consistency of fresh UHPFRC with WMS and its smaller particle size favours the coMPactness of the concrete and causes an increase in compressive strength around 11% for all substitution percentages.

There is a slight decrease in the modulus of elasticity, which does not reach 6% for a 100% substitution with WMS, so the influence of using this type of waste on the modulus of elasticity of UHPFRC is negligible.

The results of flexural strength and tensile strength obtained show high variability for each substitution percentage. This may be a consequence of an inefficient distribution of the short steel fibres present in these concretes. However, the variations are small up to 70% substitution. Only for substitutions of 100% is there a significant loss in flexural strength and tensile strength, which reach values of 17% and 20%, respectively.

It can be held that the use of up to 70% of waste mine sand as a substitute for natural sand is a totally feasible option for manufacturing UHPFRC, given that it has a minimal effect on any of its mechanical properties. However, for a ratio of 100% of substitution there is a drop of over 15% in tensile and flexural strengths, making the use of this substitution percentage inadvisable.

## Figures and Tables

**Table 1:** Properties of the materials.

Properties	Aggregate 0–0.5 mm	Aggregate 0.5–1.6 mm	Micronized Quartz	Silica Fume	Waste Mining Sand
Bulk density (kg/m^3^)	2616	2616	2609	2300	2717
Sand equivalent	97	97	-	-	28
Absorption 24 h	0.28%	0.53%	-	-	-
Humidity (%)	0%	0%	< 0.2%	< 3.00%	0%

**Table 2:** Chemical analysis (%) of the waste mining sand and the natural sand it replaces.

Material	SiO_2_	CaO	Fe_2_O_3_	Al_2_O_3_	MgO	K_2_O	Na_2_O	TiO_2_	P_2_O_5_	MnO	L.O.I
Waste mining sand	71.65	10.8	1.66	1.5	1.21	0.57	0.13	0.11	0.03	0.01	8.82
Aggregate 0–0.5 mm	99.34	< 0.1	< 0.13	0.30	< 0.1	< 0.1	< 0.03	< 0.1	< 0.1	-	0.23

**Table 3:** Dosages of Ultra High Performance Fibre Reinforced Concrete (UHPFRC) (kg/m^3^).

Material	Control	50% WMS	70% WMS	100% WMS
Cement	800	800	800	800
Sand 0–0.5 mm	302	151	91	-
Waste mining sand	-	161	225	321
Sand 0.5–1.6 mm	565	565	565	565
Micronized quartz	225	225	225	225
Silica fume	175	175	175	175
Water	175	175	175	175
Superplasticizer	10	10	10	10
Steel fibres	160	160	160	160

**Table 4:** Results of the experimental program.

Properties	Control	Difference (%)		
		50% WMS	70% WMS	100% WMS
Slump (cm)	25.0	16.0%	4.0%	20.0%
Density (kg/m^3^)	2410.0	−1.2%	−1.7%	−2.1%
Compressive strength (MPa)	117.2	9.9%	11.8%	11.2%
Modulus of elasticity (GPa)	45.2	−3.5%	−5.8%	−6.4%
Flexural strength (MPa)	23.0	−7.0%	−3.5%	−17.4%
Tensile strength (MPa)	8.7	9.2%	−1.1%	−20.7%
